# Supplementary material for: Respiratory long COVID in aged hamsters features impaired lung function post-exercise with bronchiolization and fibrosis
Source: Nat Commun. 2025 Feb 28;16:2080. doi: 10.1038/s41467-025-57267-x (PMC11871369; doi:10.1038/s41467-025-57267-x)
Supplement: Supplementary file 1 — Supplementary Information [file 41467_2025_57267_MOESM1_ESM.pdf]

Respiratory long COVID in aged hamsters features impaired lung function post-exercise with bronchiolization and fibrosis

Laura Heydemann[1,#], Małgorzata Ciurkiewicz[1,#], Theresa Störk[1], Isabel Zdora[1], Kirsten Hülskötter[1], Katharina Manuela Gregor[1], Lukas Mathias Michaely[1], Wencke Reineking[1], Tom Schreiner[1], Georg Beythien[1], Asisa Volz[2,4], Tamara Tuchel[2,4], Christian Meyer zu Natrup[2,4], Lisa-Marie Schünemann[2,4], Sabrina Clever[2,4], Timo Henneck[3,4], Maren von Köckritz-Blickwede[3,4], Dirk Schaudien[5], Karl Rohn[6], Klaus Schughart[7,8], Robert Geffers[9], Mika K. Kaneko[10], Yukinari Kato[10], Carina Gross[11], Georgios Amanakis[11], Andreas Pavlou[12], Wolfgang Baumgärtner[1,#,§] and Federico Armando[1,13,#]

[1]Department of Pathology, University of Veterinary Medicine, Foundation, Hanover, Germany
[2]Department of Virology, University of Veterinary Medicine, Foundation, Hanover, Germany
[3]Department of Biochemistry, University of Veterinary Medicine, Foundation, Hanover, Germany
[4]Research Center for Emerging Infections and Zoonoses (RIZ), University of Veterinary Medicine, Foundation, Hanover, Germany
[5]Fraunhofer Institute for Toxicology and Experimental Medicine (ITEM), Hanover, Germany
[6]Department of Biometry, Epidemiology and Data Management, University of Veterinary Medicine, Foundation, Hanover, Germany
[7]Department of Microbiology, Immunology and Biochemistry, University of Tennessee Health Science Center, Memphis, Tennessee, USA,
[8]Institute of Virology Münster, University of Münster, Münster, Germany
[9]Helmholtz Centre for Infection Research (HZI), Brunswick, Germany
[10]Department of antibody drug development, Tohoku University, Sendai, Miyagi, Japan
[11]Department of Cardiology, Hanover Medical School (MHH), Hanover, Germany
[12]Institute for Experimental Infection Research, TWINCORE, Centre for Experimental and Clinical Infection Research, a joint venture between the Helmholtz Centre for Infection Research and the Hannover Medical School, 30625, Hannover, Germany
[13]Pathology unit, Department of Veterinary Science, University of Parma, Parma, Italy
[#]These authors contributed equally
[§]Corresponding author

Correspondence and requests for materials should be addressed to Wolfgang Baumgärtner (Wolfgang.baumgaertner@tiho-hannover.de)

SUPPLEMENTARY DATA

This file contains:

Supplementary Tables: 3

Supplementary Figures: 11

Supplementary Table 1: Marker gene list of main pulmonary cell populations in the hamster species used in the different investigations

ADI CELLS	CLUB CELLS	AIRWAY BASAL CELLS	PRO-FIBROTIC
Anxa3	Pigr	Krt5	Eln
S100a6	Ifitm2	Krt14	Clu
Krt7	Gss	Ngfr	Flt1
Krt8	Scgb3a2	Pou2f3	Col5a2
Dstn	Hp		Col3a1
Anxa1	Fam216b		Tgfb1
Tuft1	Muc5ac		Fgf2
Tacstd2	Scgb3a1		Bmp6
Klf6			Stat3
Lmo7			Col1a1
Cdkn1a			Col1a2
Tp53			Col5a1
Tnip3			Col6a3
Hbegf			Col8a1
Ggh			Timp1
Steap4			Mmp11
Zfp36			Mmp14
Junb			Mmp12
Jun			Mmp19
Fos			Ccn2
Ndnf			Junb
Timp2			Jund
Emp2			Actb
Sox4			Acta2
Wwtr1			S100a6
Sparc			Flt3
			Vegfa
			Vegfb
			Vegfc
			Vegfd

Abbreviations: alveolar differentiation intermediate cells (ADI)

Supplementary Table 2: Description and unit of the parameters chosen for the lung function analysis using whole body plethysmography with a gas analyzer

Parameter	Description	Unit
**f**	Respiratory rate	breaths per minute (BPM)
**Tv**	Tidal volume	ml
**Te**	Expiratory time	s
**Ti**	Inspiratory time	s
**EF50**	Mid-expiratory flow (expiratory flow at 50% exhaled volume)	ml/s
**vO_2**	O_2 consumption	ml/min
**vCO_2**	CO_2 production	ml/min
**MR**	Metabolic rate: energy expenditure due to metabolism. A function of vO_2 and vCO_2	cal/min
**RQ**	Respiratory quotient: ratio of vCO_2/ vO_2. indicator of lipid and carbohydrate metabolism balance	/

References:

Menachery VD, Gralinski LE, Baric RS, Ferris MT. New Metrics for Evaluating Viral Respiratory Pathogenesis. PLoS One. 2015 Jun 26;10 (6):e0131451. doi: 10.1371/journal.pone.0131451. PMID: 26115403; PMCID: PMC4482571.

Supplementary Table 3: Primary antibodies, visualization method, dilution, clonality and host species, secondary antibody as well as positive controls used for immunohistochemical and immunofluorescence investigations.

Primary antibody	Visualization method	Dilution		Clonality, host species	Secondary antibody (1:200)		Positive control
		IHC	IF		IHC	IF	
**CK8** (Invitrogen PA-29607)	ABC	1 : 500		polyclonal, rabbit	GAR-b		Airways (Hm internal control)
**CK8-Alexa Fluor 488 conjugated** (abcam, AB192467)	/	/	1 : 100	monoclonal, rabbit, clone EP1628Y	/	/	Airways (Hm internal control)
**SCGB1A1** (Proteintec, 10490-1-AP)	ABC	1 : 1600		polyclonal, rabbit	GAR-b		Airways (Hm internal control)
**SCGB1A1-Alexa Fluor 488-conjugated** (Proteintec CL488-10490)	/	/	1 : 200	polyclonal, rabbit	/	/	Airways (Hm internal control)
**CK14** (invitrogen, PA5-16722)	ABC	1 : 500		polyclonal, rabbit	GAR-b		Airways (Hm internal control)
**CK14** (invitrogen, MA5-11599)	/	/	1 : 250	monoclonal, mouse, clone LL002		GAM-Cy3	Airways (Hm internal control)
**proSP-C** (MEMD Millipore, AB3786)	/	/	1 : 1000	polyclonal, rabbit	/	GAR-Cy5	AT2
**Pdpn** (kindly provided by *Prof. Kato, Tohoku University, Japan*)	/	/	1 : 400	monoclonal, mouse, 281-mG2a-f	/	GAM-Cy3	AT1
**p53** (NovusBiologicals, BP53-12)	ABC	1 : 200	/	monoclonal, mouse, clone BP53-12	GAM-b	/	Lymph node (Hm)
**Ki-67** (abcam, ab15580-100)	ABC	1 : 500	1 : 1000	polyclonal, rabbit	GAR-b	GAR-Cy3 GAR-Cy5	Lymph node (Hm)
**p21** (SantaCruz Biotechnology,	ABC	1 : 100	/	monoclonal	GAM-b	/	Lymph node (Hm)

sc6246)				, mouse, F-5			
**Δnp63** (Cell Signaling #67825)	ABC	1 : 1000	/	monoclonal, rabbit, clone E6Q30	GAR-b	/	Oral squamous epithelia (Hm)
**CD3** (Dako, A0452)	ABC	1 : 500	/	polyclonal, rabbit	GAR-b	/	SARS-CoV-2 infected lung, 6dpi (Hm internal control)
**pax5** (BioLegend, Cat649702)	ABC	1 : 500	/	monoclonal, rat, 1H9	RaRat-b	/	SARS-CoV-2 infected lung, 6dpi (Hm internal control)
**Iba-1** (ThermoFisher, PA5-27436)	ABC	1 : 1000	/	polyclonal, rabbit	GAR-b	/	SARS-CoV-2 infected lung, 6dpi (Hm internal control)
**Plet-1** (biorbyt, orb312797_1)	ABC	1 : 200	/	polyclonal, rabbit	GAR-b	/	alveolar macrophages
**MPO** (abcam, ab9535)	ABC	1 : 200	/	rabbit, polyclonal	GAR-b	/	SARS-CoV-2 infected lung, 6dpi (Hm internal control)
**CD204** (Abnova Corporation, MAB1710)	ABC	1 :500	/	monoclonal, mouse, clone SRA-E5	/	/	SARS-CoV-2 infected lung, 6dpi (Hm internal control)
**SARS-CoV-2 NP** (Sinobiological, 40143-MM05)	EnVision	1 : 16000	/	monoclonal, mouse, clone 5	/	/	SARS-Cov-2 infected lung, 3 dpi (Hm)
**SARS-CoV-2 SP** (Sino Biological Inc., HD14AP0706)	ABC	1 : 4000	/	polyclonal, rabbit	GAR-b	/	SARS-Cov-2 infected lung, 3 dpi (Hm)

Abbreviations: AT1 = alveolar epithelial cell type 1, AT2 = alveolar epithelial cell type 2, -b = biotinylated, CD3 = cluster of differentiation 3, CD204 = cluster of differentiation 204, CK8 = Cytokeratin 8, CK14 = Cytokeratin 14, GAM = goat-anti-mouse, GAR = goat-anti-rabbit, Hm = hamster, Iba -1 = ionized calcium-binding adapter molecule 1, IHC = immunohistochemistry, IF = immunofluorescence, Ki-67 = antigen Kiel 67, MPO = myeloperoxidase, pax5 = paired box protein 5, Pdpn = Podoplanin, p53 = tumor protein 53, RaRat = rabbit-anti-rat, SARS-CoV-2 NP = SARS-CoV-2 nucleocapsid protein, SARS-CoV-2 SP= SARS-CoV-2 Spike protei,n proSP-C = pro-surfactant protein C, SCGB1A1 = secretoglobin 1a1, Plet-1 =placenta expressed transcript-1

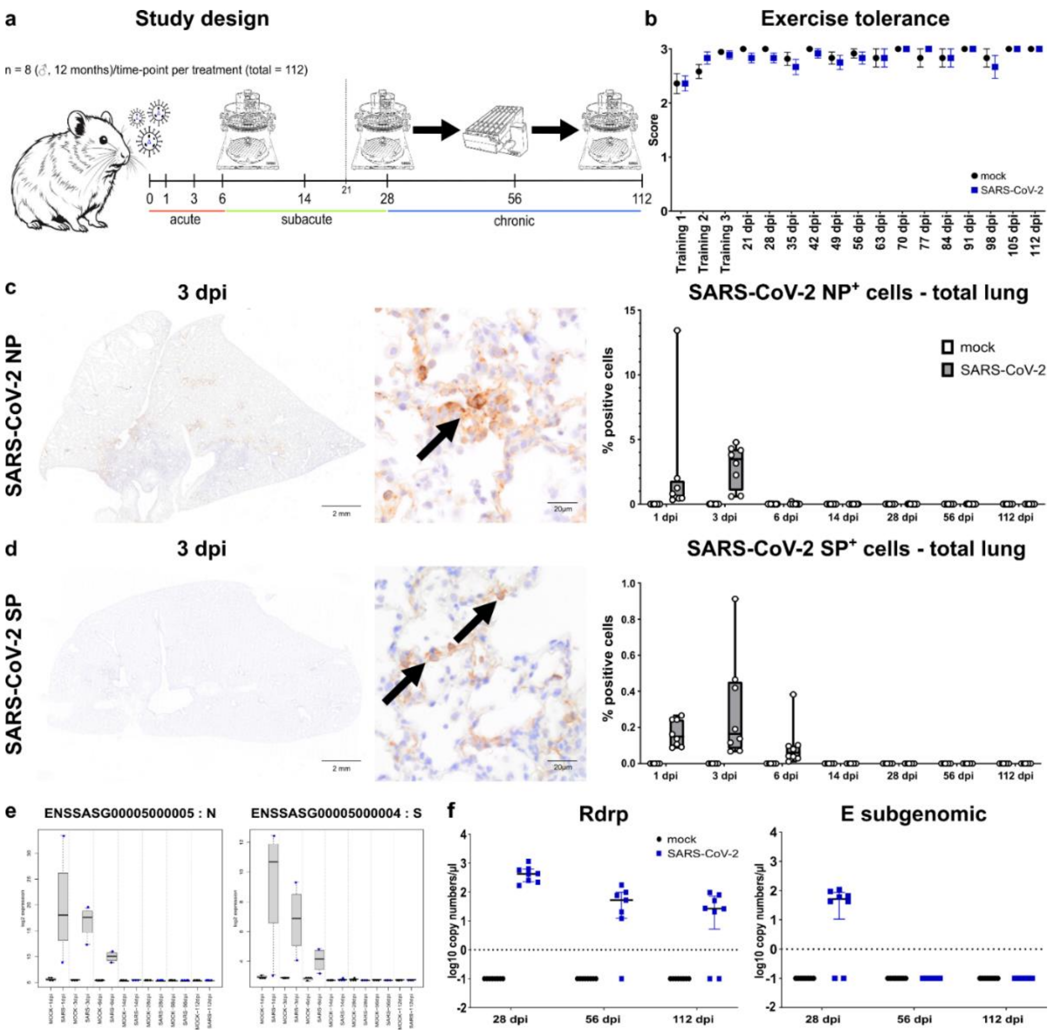

Supplementary figure 1: Experimental design, exercise tolerance and virus quantification

a Schematic drawing of the experimental design. Male, 1-year old Syrian hamsters were infected with SARS-CoV-2 Delta variant and sacrificed at 1, 3, 6, 14, 28, 56 and 112 dpi. The study design allowed to distinguish three phases of the disease: acute phase (infection – 6 dpi), sub-acute phase (6 – 28 dpi), and chronic phase (28 dpi – 112 dpi). During the experiment, repeated lung function measurements were conducted using whole-body plethysmography (WBP) with respiratory gas analysis. Physical exercise on a rodent treadmill was used to exacerbate possible latent respiratory impairment from 21 dpi onwards and was repeated weekly. **b** Scoring of exercise tolerance (running behavior on the treadmill, mean and SEM). $N = 18$ (Training 1 until 28 dpi), 12 (35-56 dpi), or 6 (63-112 dpi) animals/group. **c-d** Immunohistochemistry for SARS-CoV-2 nucleoprotein (NP, c), and spike protein (SP, d). For each staining, an overview, a high magnification and the quantification in the whole section of the left lung lobe is shown. Quantitative data is shown as box and whisker plots. The bounds of the box plot indicate the 25th and 75th percentiles, the bar indicates medians, and the whiskers indicate minima and maxima. Dots show individual values. Data was tested by two-tailed Mann–Whitney *U* test. A p value of ≤0.05 was chosen as the cutoff for statistical significance. $N = 8$ animals/group/time-point. **e** Beeswarm plots for SARS-CoV-2 genes. The bounds of the box plot indicate the 25th and 75th percentiles, the bar indicates medians, and the whiskers indicate minima and maxima. Dots show individual values. $N = 4$ animals/group/time-point. **f** PCR for SARS-CoV-2 RNA-dependent RNA polymerase (RdRp) and subgenomic viral RNA in the chronic phase. Graphs show mean, SEM, and individual values. $N = 8$ animals/group/time-point. Negative results were set to -1 to enhance readability. Source data is provided as a Source Data file.

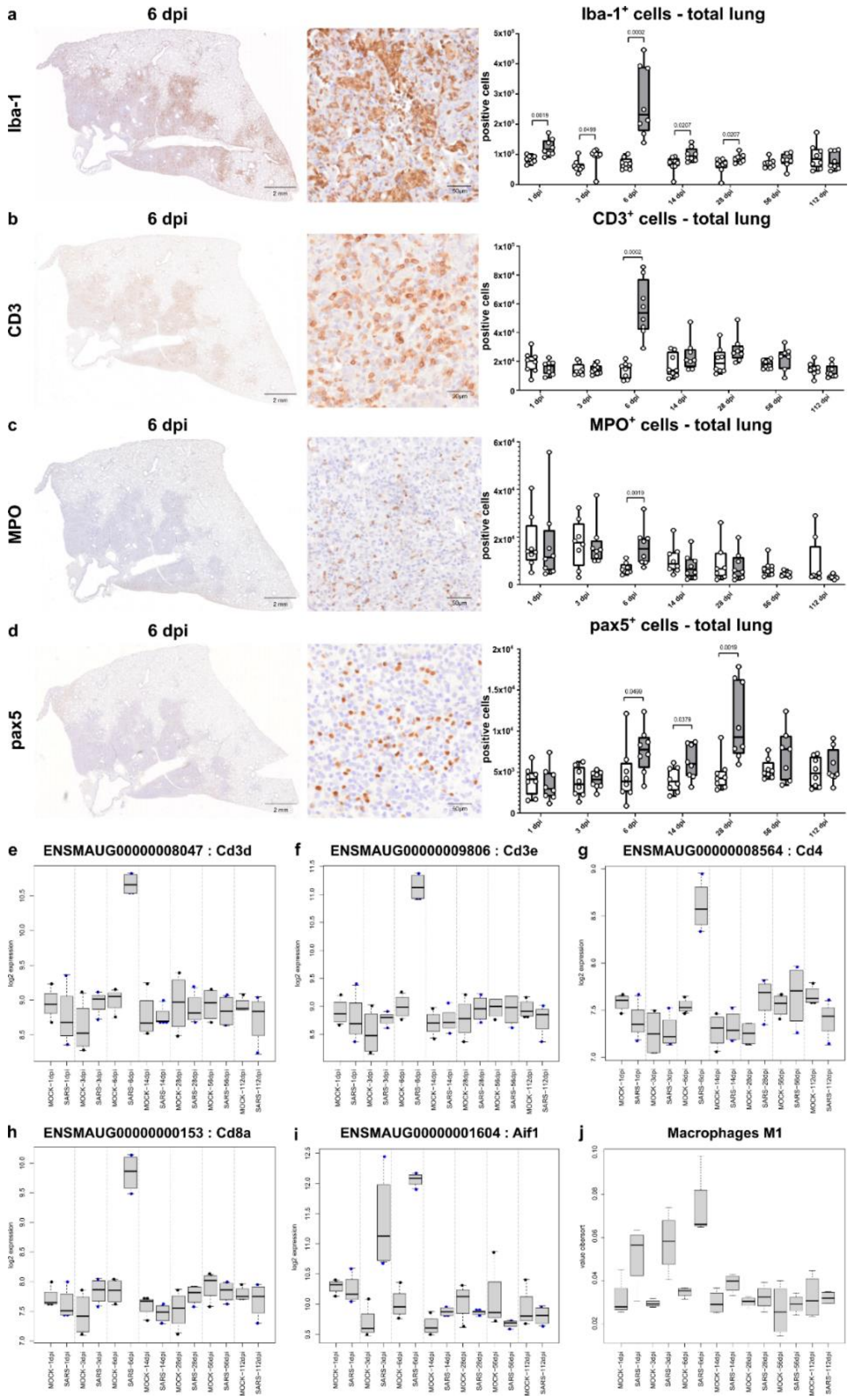

Supplementary figure 2: Inflammatory cells in the lung.

a-d Immunohistochemistry for Iba-1 (macrophages, a), CD3 (T cells, b), myeloperoxidase (MPO, heterophils, c), and Pax5 (B cells, d). For each staining, an overview, a high magnification and the quantification in the whole section of the left lung lobe is shown. **e-j** Beeswarm plots for T-cell genes (e-h), and macrophage gene (i). In f there is a cybersortX deconvolution for M1-like macrophage signature genes. Quantitative data is shown as box and whisker plots. The bounds of the box plot indicate the 25th and 75th percentiles, the bar indicates medians, and the whiskers indicate minima and maxima. Dots indicate individual values. In a-d, data was tested by two-tailed Mann–Whitney *U* test. A *p* value of ≤0.05 was chosen as the cutoff for statistical significance. *N* = 8 animals/group/time-point (a-d) or 4 animals/group/time-point (e-j). Source data is provided as a Source Data file.

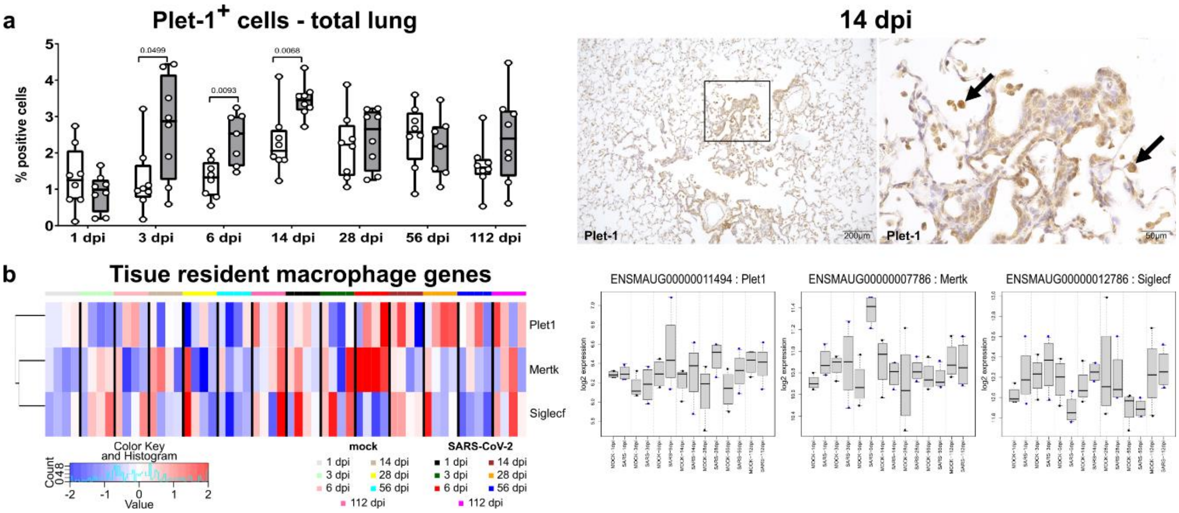

Supplementary figure 3: T cells and macrophage-epithelial cross-talk.
a Immunohistochemistry for Placenta expressed transcript-1 (Plet-1), a mediator of macrophage-epithelial crosstalk in alveolar proliferation. Quantification in the whole section of the left lung lobe, a low and a high magnification is shown. Positive alveolar macrophages are indicated by arrows. **b** Heatmap and beeswarms for *Plet1* and related genes. In a, b, and d, expression values are scaled by row. Red indicates higher and blue lower relative expression levels. In c, and d, data is shown as box and whisker plots. The bounds of the box plot indicate the 25th and 75th percentiles, the bar indicates medians, and the whiskers indicate minima and maxima. Dots indicate individual values. In c, data was tested by two-tailed Mann–Whitney U test. A p value of ≤0.05 was chosen as the cutoff for statistical significance. N = 8 animals/group/time-point (c) or 4 animals/group/time-point (a, b, d). Source data is provided as a Source Data file.

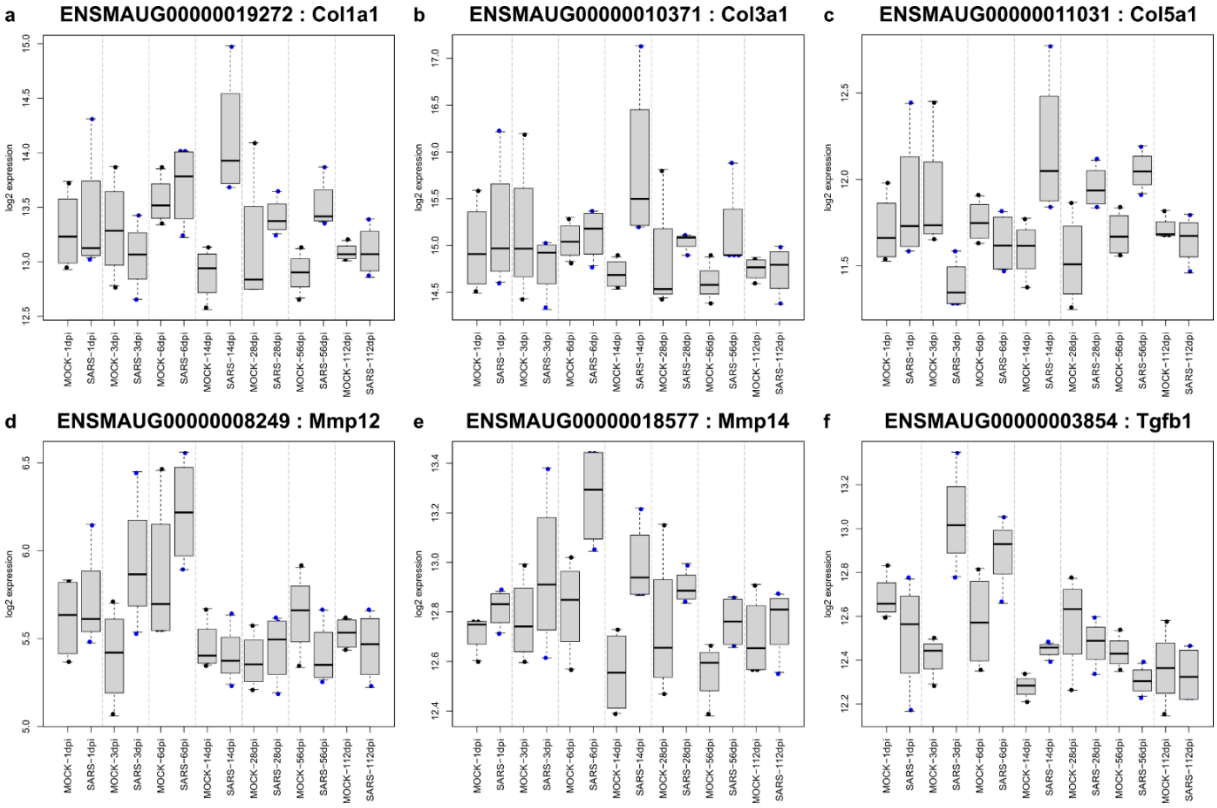

Supplementary figure 4: Pro-fibrotic environment.

Beeswarm plots for genes associated with a pro-fibrotic environment in mock- and SARS-CoV-2 infected hamsters at different time-points post infection. The bounds of the box plot indicate the 25th and 75th percentiles, the bar indicates medians, and the whiskers indicate minima and maxima. Dots indicate individual values. *N* = 4 animals/group/time-point.

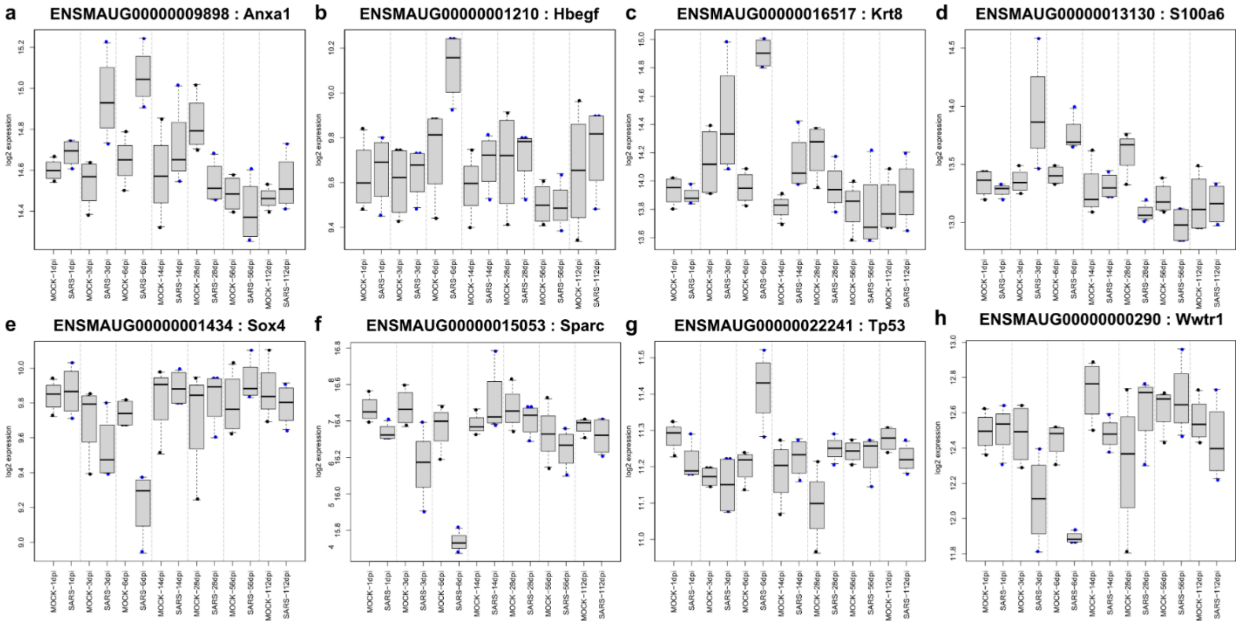

Supplementary figure 5: alveolar differentiation intermediate (ADI) cells. Beeswarm plots for ADI cell genes in mock- and SARS-CoV-2 infected hamsters at different time-points post infection. The bounds of the box plot indicate the 25th and 75th percentiles, the bar indicates medians, and the whiskers indicate minima and maxima. Dots indicate individual values. *N* = 4 animals/group/time-point.

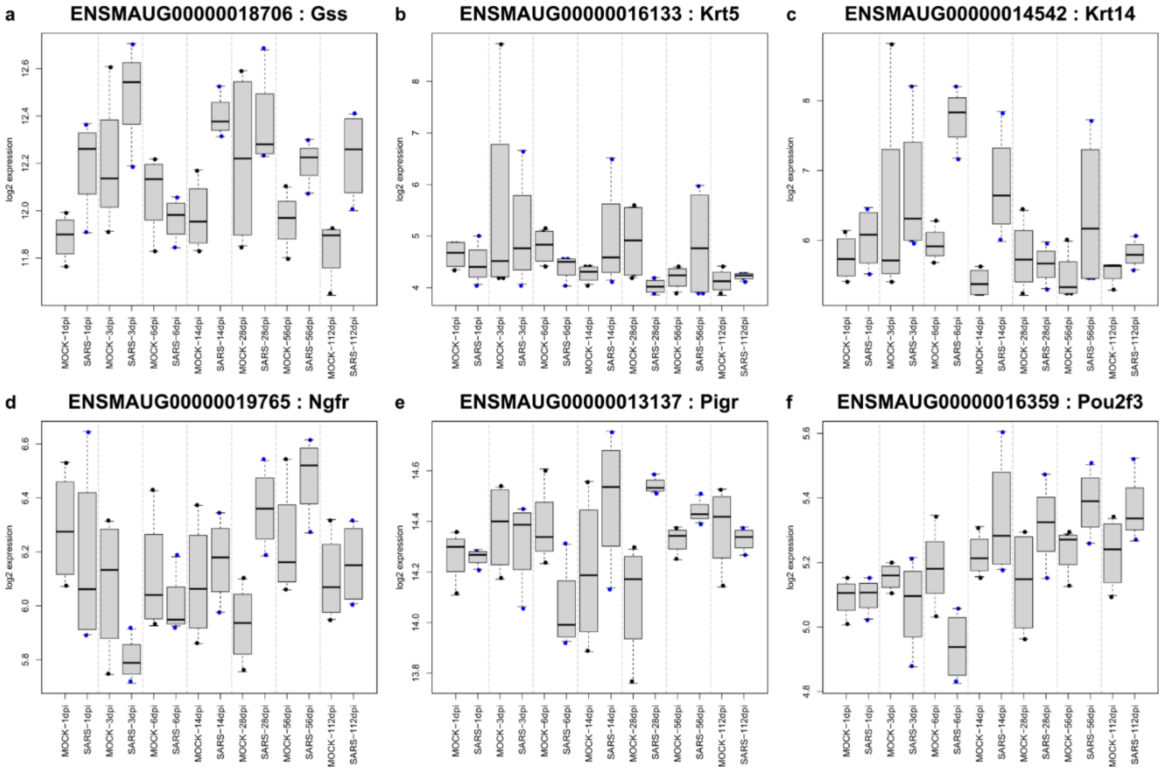

Supplementary figure 6: airway cells.

Beeswarm plots for airway basal cell genes (a-c) and club cell genes (d-f) in mock- and SARS-CoV-2 infected hamsters at different time-points post infection. The bounds of the box plot indicate the 25th and 75th percentiles, the bar indicates medians, and the whiskers indicate minima and maxima. Dots indicate individual values. *N* = 4 animals/group/time-point.

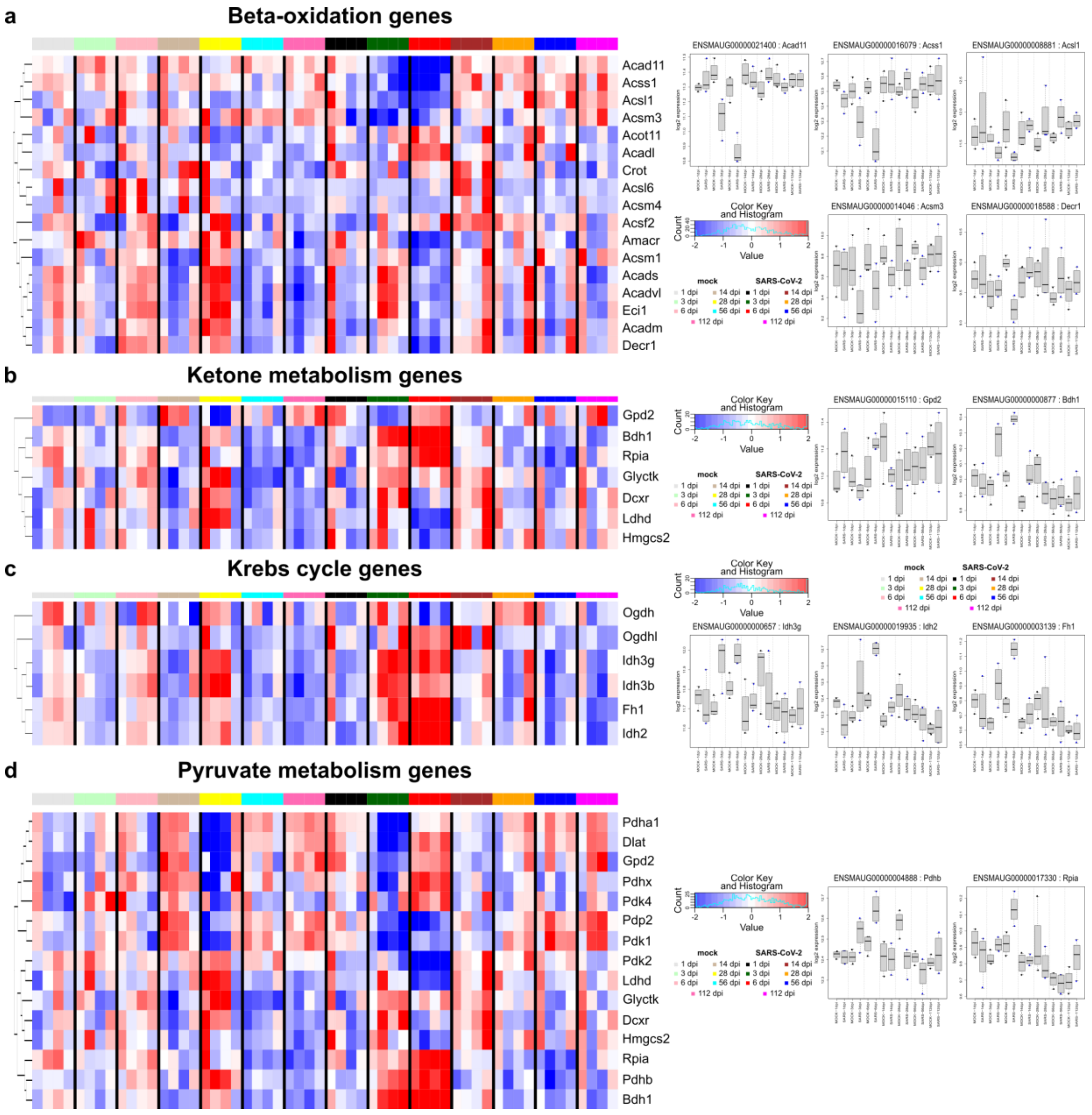

Supplementary figure 7: Metabolism.
Heatmaps of normalized expression values and beeswarm plots of selected genes involved in different metabolic pathways at each dpi in mock- and SARS-CoV-2-infected hamsters. In the heatmaps, expression values are scaled by row. Red indicates higher and blue lower relative expression levels. In the beeswarm plots, the bounds of the box plot indicate the 25th and 75th percentiles, the bar indicates medians, and the whiskers indicate minima and maxima. Dots indicate individual values. *N* = 4 animals/group/time-point.

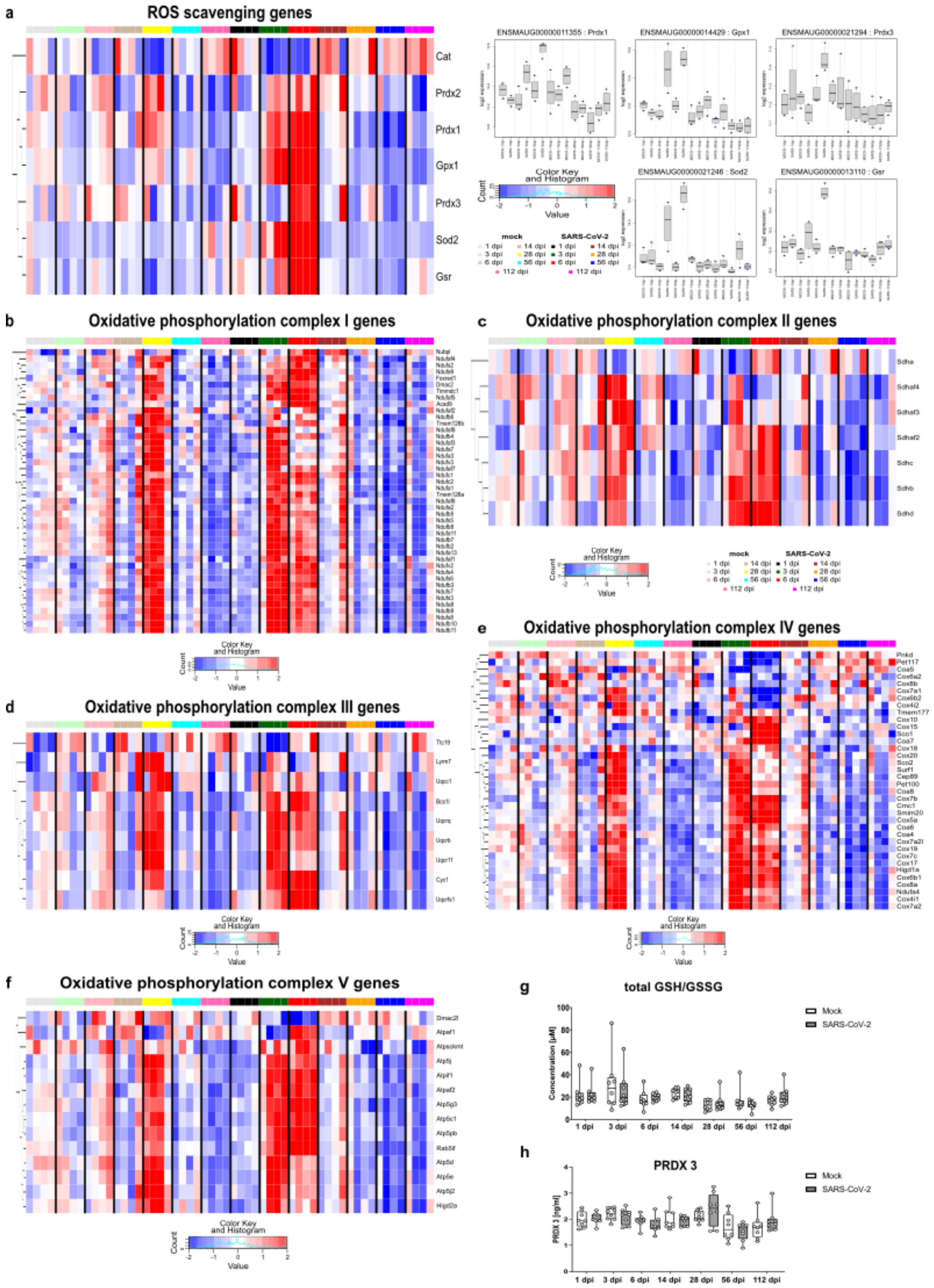

Supplementary figure 8: Mitochondrial Oxidative phosphorylation.
a-f Heatmaps of normalized expression values and beeswarm plots of selected genes involved in ROS scavenging and oxidative phosphorylation at each dpi in mock- and SARS-CoV-2- infected hamsters. In the heatmaps, expression values are scaled by row. Red indicates higher and blue lower relative expression levels. In the beeswarm plots, the bounds of the box plot indicate the 25th and 75th percentiles, the bar indicates medians, and the whiskers indicate minima and maxima. Dots indicate individual values. *N* = 4 animals/group/time-point. **g, h** Quantification of total Glutathione (GSH/GSSG, g) and Peroxiredoxin 3 (Prdx3, h) in serum samples of mock- and SARS-CoV-2-infected hamsters. Data is shown as box and whisker plots. The bounds of the box plot indicate the 25th and 75th percentiles, the bar indicates the median, and the whiskers indicate minima and maxima. Dots indicate individual values. In c, data was tested by two-tailed Mann–Whitney U test. A p value of ≤0.05 was chosen as the cutoff for statistical significance. N = 8 animals/group/time-point

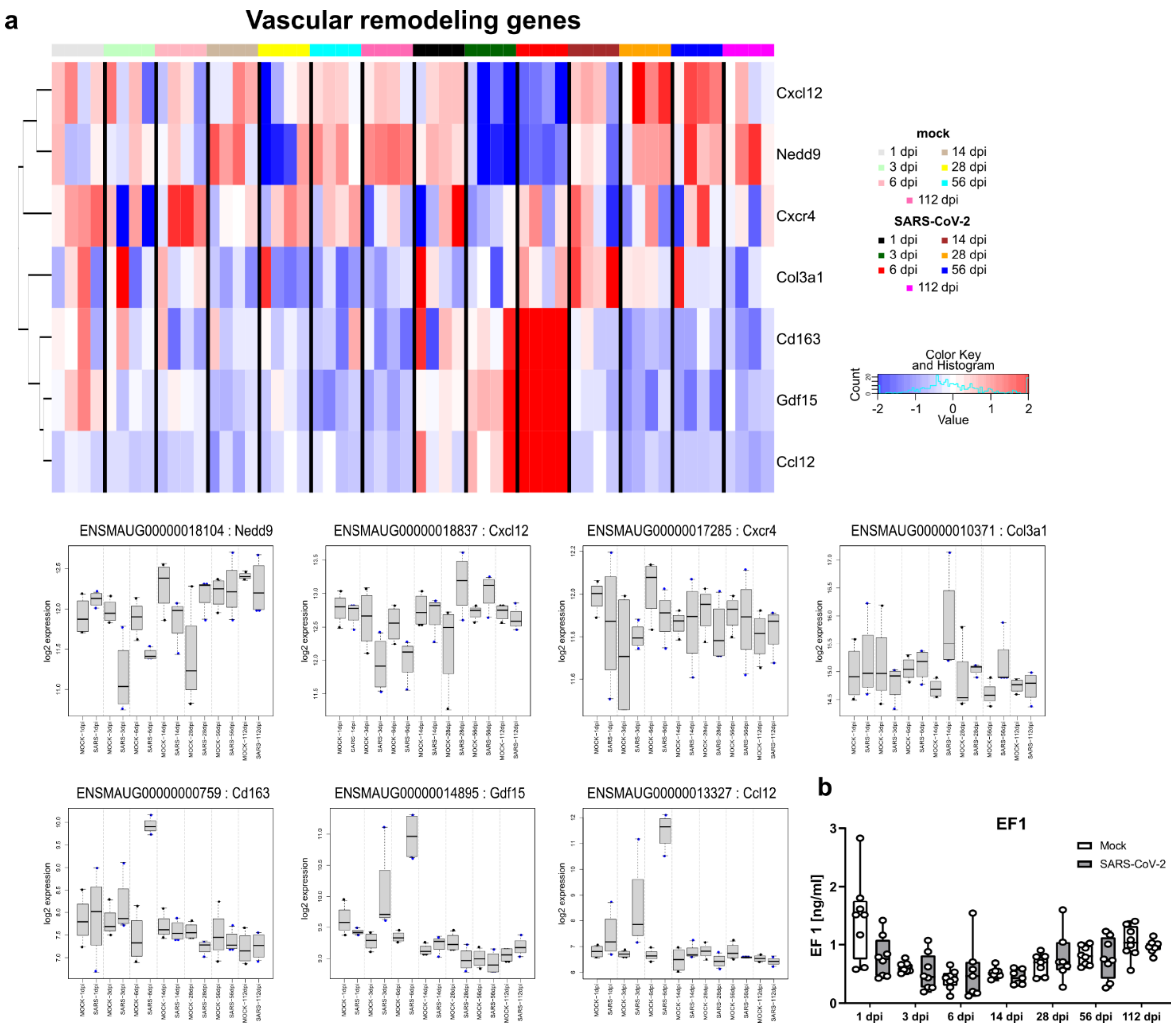

Supplementary figure 9: Vascular remodeling.

a Heatmaps of normalized expression values and beeswarm plots of genes involved in COVID-19-associated pulmonary vascular remodeling at each dpi in mock- and SARS-CoV-2-infected hamsters. In the heatmaps, expression values are scaled by row. Red indicates higher and blue lower relative expression levels. In the beeswarm plots, the bounds of the box plot indicate the 25th and 75th percentiles, the bar indicates medians, and the whiskers indicate minima and maxima. Dots indicate individual values. *N* = 4 animals/group/time-point. **b** Quantification of EF1, a marker for COVID-19-associated vascular dysfunction, in the serum of mock- and SARS-CoV-2-infected hamsters. Data is shown as box and whisker plots. The bounds of the box plot indicate the 25th and 75th percentiles, the bar indicates medians, and the whiskers indicate minima and maxima. Dots indicate individual values. Data was tested by two-tailed Mann–Whitney U test. A p value of ≤0.05 was chosen as the cutoff for statistical significance. *N* = 8 animals/group/time-point.

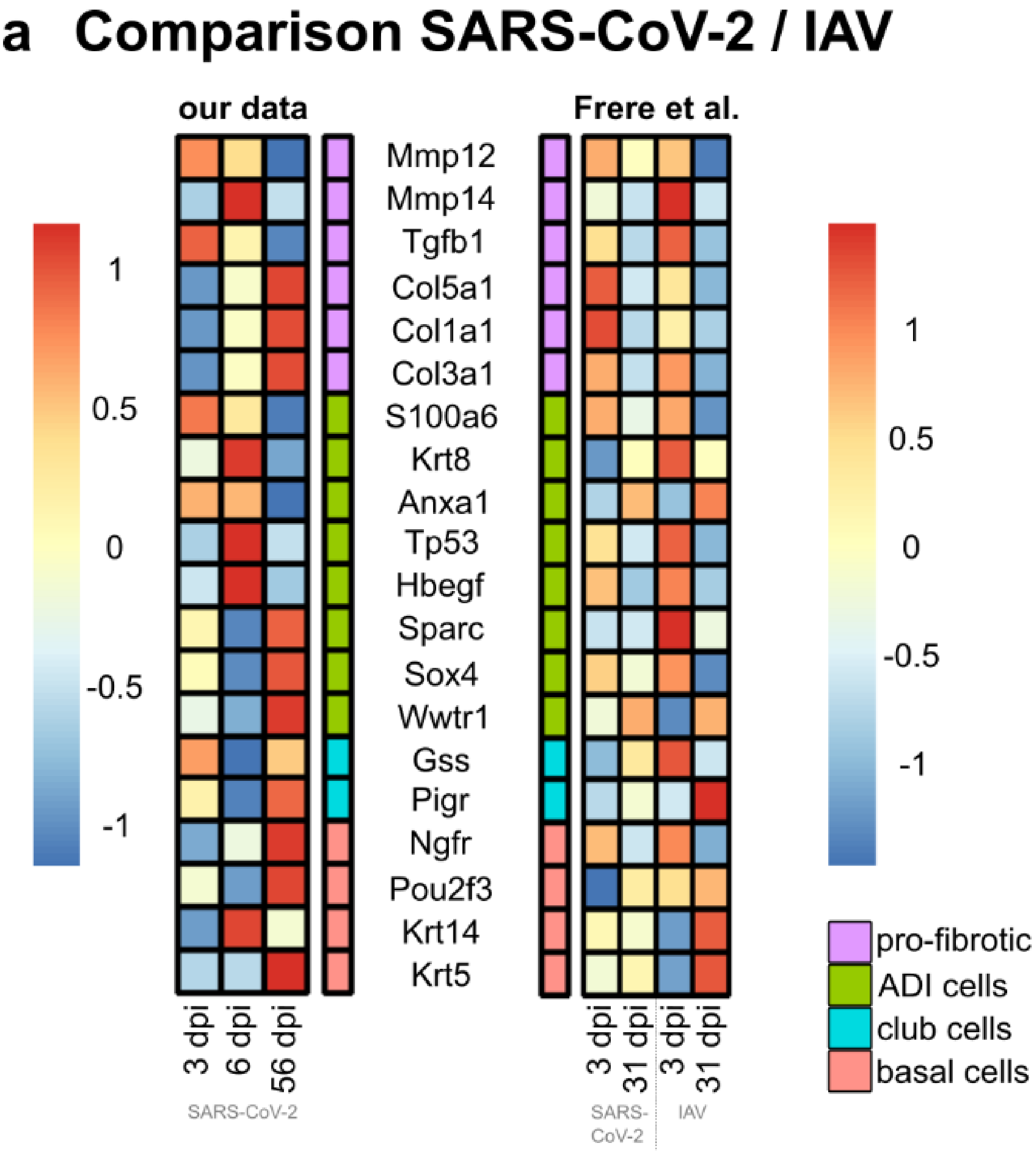

Supplementary figure 10: comparison SARS-CoV-2 and IAV-infection in hamsters.

Genes assocciated with a pro-fibrotic environment, ADI, club, or basal cells expressed in the lung of SARS-CoV-2 infected hamsters in this experiment were selected and the expression pattern was compared with a published dataset obtained in SARS-CoV-2- and IAV-infected hamsters (Frere et al., 2022). Heatmaps (pheatmap) show $\log_2$ fold changes of normalized expression values (means per group) to the respective mock control means. Expression values are scaled by row. Red/orange indicates higher and blue/lightblue lower relative expression levels.

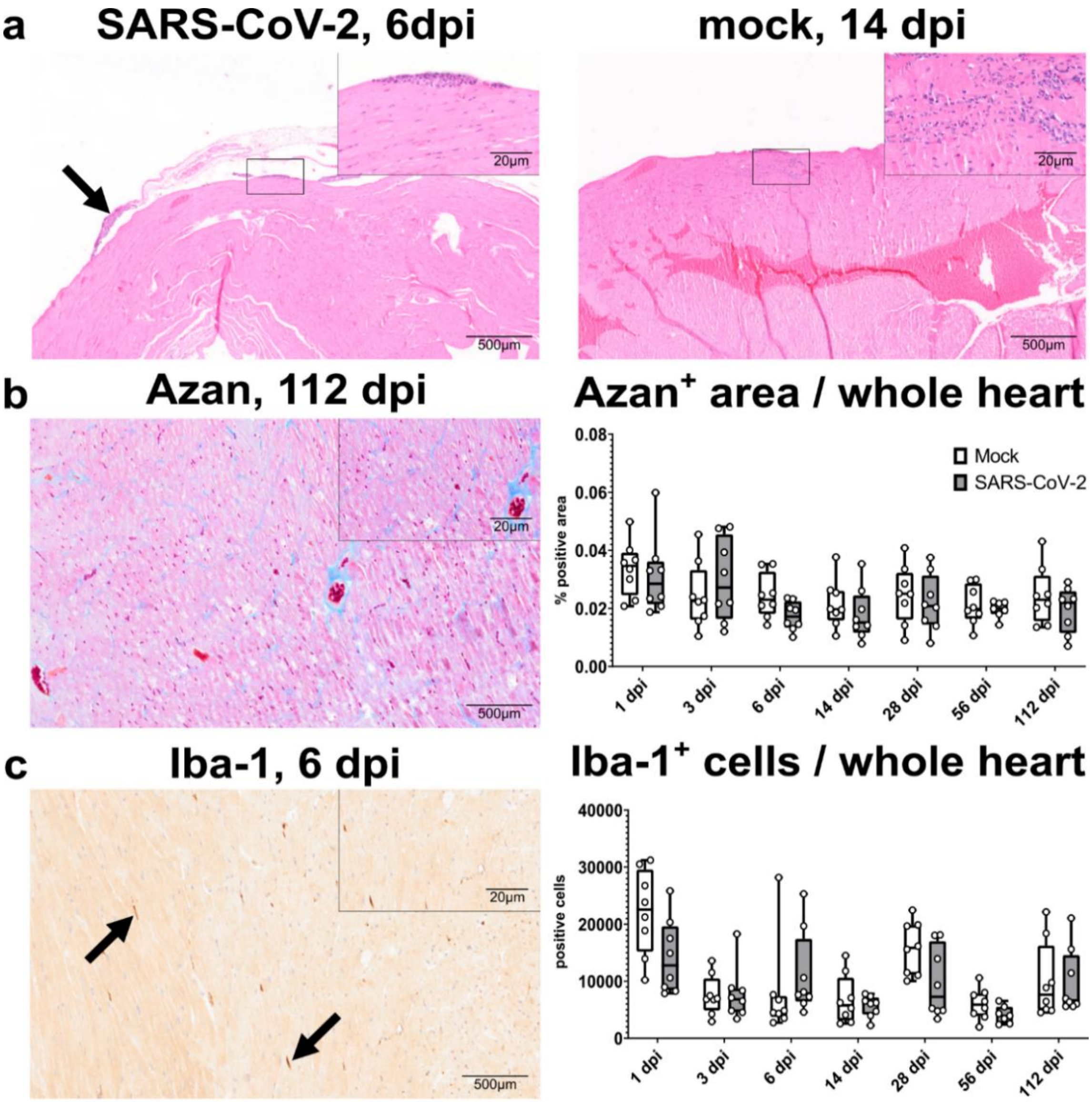

Supplementary figure 11: SARS-CoV-2 infection does not cause marked long term effects in the heart, kidney, liver or spleen of aged hamsters.

a-c Lack of SARS-CoV-2 infection-related lesions in the hearts of hamsters. a Mild subepicardial inflammation was detected in SARS-CoV-2 (left panel) and mock (right panel) infected animals. Hematoxylin and eosin (HE) stain. b Left: Mild myocardial fibrosis (blue staining, Azan stain) in a SARS-CoV-2 infected hamster. Right: Quantification of Azan positive area in the hearts of mock and SARS-CoV-2 infected hamsters shows no difference between the groups. c Right: Immunohistochemistry showing small numbers of Iba-1$^+$ histiocytic cells (arrows) in the heart of a SARS-CoV-2 infected hamster. Right: Quantification of Iba-1$^+$ cells in the hearts of mock and SARS-CoV-2 infected hamsters shows no difference between the groups.
