# Supplementary material for: Respiratory long COVID in aged hamsters features impaired lung function post-exercise with bronchiolization and fibrosis
Source: Nat Commun. 2025 Feb 28;16:2080. doi: 10.1038/s41467-025-57267-x (PMC11871369; doi:10.1038/s41467-025-57267-x)
Supplement: Supplementary file 2 — Description of Additional Supplementary Files [file 41467_2025_57267_MOESM2_ESM.docx]

**File name: Supplementary Data 1**

**Description:** Tables with all the clinical data collected during the experiment

This table is submitted as separate excel file.

**File name: Supplementary Data 2**

**Description:** list of differentially expressed genes (DEGs) per every time-point

This table is submitted as separate excel file.

**File name: Supplementary Data 3**

**Description:** list of differentially expressed genes (DEGs) unique to the sub-acute and chronic phase of the disease

This table is submitted as separate excel file.

**File name: Supplementary Data 4**

**Description:** Table with all the semi-quantitative histopathological scores of the lung

This table is submitted as separate excel file.

**File name: Supplementary Data 5**

**Description:** Table with all the histopathological evaluation of the heart

This table is submitted as separate excel file.

**File name: Supplementary Data 6**

**Description:** Table with all the histopathological evaluation and SARS-CoV-2 immunolabellings of the kidney, liver and spleen. In addition there are semi-quantitative scores for Azan staining and immunolabellings for Iba-1, CD3 and MPO (kidney)

This table is submitted as separate excel file.

**File name: Supplementary Data 7**

**Description:** list of analyzed genes involved in different mitochondrial pathways or vascular remodeling
