# Supplementary material for: Respiratory long COVID in aged hamsters features impaired lung function post-exercise with bronchiolization and fibrosis
Source: Nat Commun. 2025 Feb 28;16:2080. doi: 10.1038/s41467-025-57267-x (PMC11871369; doi:10.1038/s41467-025-57267-x)
Supplement: Supplementary file 10 — Reporting Summary [file 41467_2025_57267_MOESM10_ESM.pdf]

nature portfolio

Corresponding author(s): Wolfgang Baumagaertner

Last updated by author(s): Jan 27, 2025

Reporting Summary

Nature Portfolio wishes to improve the reproducibility of the work that we publish. This form provides structure for consistency and transparency in reporting. For further information on Nature Portfolio policies, see our Editorial Policies and the Editorial Policy Checklist.

Statistics

For all statistical analyses, confirm that the following items are present in the figure legend, table legend, main text, or Methods section.

n/a	Confirmed	
☐	☒	The exact sample size (*n*) for each experimental group/condition, given as a discrete number and unit of measurement
☐	☒	A statement on whether measurements were taken from distinct samples or whether the same sample was measured repeatedly
☐	☒	The statistical test(s) used AND whether they are one- or two-sided<br>*Only common tests should be described solely by name; describe more complex techniques in the Methods section.*
☒	☐	A description of all covariates tested
☐	☒	A description of any assumptions or corrections, such as tests of normality and adjustment for multiple comparisons
☐	☒	A full description of the statistical parameters including central tendency (e.g. means) or other basic estimates (e.g. regression coefficient) AND variation (e.g. standard deviation) or associated estimates of uncertainty (e.g. confidence intervals)
☐	☒	For null hypothesis testing, the test statistic (e.g. *F*, *t*, *r*) with confidence intervals, effect sizes, degrees of freedom and *P* value noted<br>*Give P values as exact values whenever suitable.*
☒	☐	For Bayesian analysis, information on the choice of priors and Markov chain Monte Carlo settings
☒	☐	For hierarchical and complex designs, identification of the appropriate level for tests and full reporting of outcomes
☒	☐	Estimates of effect sizes (e.g. Cohen's *d*, Pearson's *r*), indicating how they were calculated

Our web collection on statistics for biologists contains articles on many of the points above.

Software and code

Policy information about availability of computer code

Data collection	lung function data: Measurements were collected with the software “FinePointe Respiratory Software” (Version 3.0.1.13370)
Data analysis	RNAseq data analysis: The reference file for golden hamster (MesAur1.0) was taken from Ensemble database release 110. The following steps of analysis were performed using R software package (version 4.2.1) and RStudio (version 2022.02.0 Build 443) . Feature counts were obtained by Rsubread v2.16.0 followed by feature annotation using biomaRt_2.54.1 In cases where the gene symbol information for hamster genes was missing, the orthologous human gene symbols were used instead. For the transcriptome analysis, only genes with a biotype of 'protein coding' and counts greater than 10 in at least four samples were included. Differential gene expression analysis was performed using DESeq2 (version 1.36.0) after normalizing gene counts with the rlog function (regularized log transformation). Volcano plots were generated with the package<br>EnhancedVolcano (version 1.14.0). Functional analyses of DEGs were performed using the R software package cluster Profiler (version 4.4.4) . Heatmaps were generated with the function heatmap2 of package gplots (version 3.1.3; https://github.com/talgalili/gplots).Upset plots were prepared with package UpSetR (version 1.4.0) . For beeswarm graphs of expression levels, package beeswarm (version 0.4.0) was used.<br>statistical analysis: graph pad prism version 9.3.1 or SAS® version 9.4<br><br>image analysis: immunohistochemistry qu path (version 0.5.0)<br><br>Image analysis: software (Visiopharm Software Version 2023.01, Hoersholm Denmark) was used to measure the Azan positive areas<br><br>lung function data: Measurements were analyzed with the software “FinePointe Respiratory Software” (Version 3.0.1.13370)

For manuscripts utilizing custom algorithms or software that are central to the research but not yet described in published literature, software must be made available to editors and reviewers. We strongly encourage code deposition in a community repository (e.g. GitHub). See the Nature Portfolio guidelines for submitting code & software for further information.

Data

Policy information about availability of data

All manuscripts must include a data availability statement. This statement should provide the following information, where applicable:

- Accession codes, unique identifiers, or web links for publicly available datasets
- A description of any restrictions on data availability
- For clinical datasets or third party data, please ensure that the statement adheres to our policy

The data generated in this study are provided in the Supplementary Information and Source Data file. Bulk RNASeq data from the lungs of SARS-CoV-2-infected hamsters are made publicly available, reference from GEO is provided: GSE271365. Source data are provided with this paper.

Research involving human participants, their data, or biological material

Policy information about studies with human participants or human data. See also policy information about sex, gender (identity/presentation), and sexual orientation and race, ethnicity and racism.

Reporting on sex and gender	*Use the terms sex (biological attribute) and gender (shaped by social and cultural circumstances) carefully in order to avoid confusing both terms. Indicate if findings apply to only one sex or gender; describe whether sex and gender were considered in study design; whether sex and/or gender was determined based on self-reporting or assigned and methods used. Provide in the source data disaggregated sex and gender data, where this information has been collected, and if consent has been obtained for sharing of individual-level data; provide overall numbers in this Reporting Summary. Please state if this information has not been collected. Report sex- and gender-based analyses where performed, justify reasons for lack of sex- and gender-based analysis.*
Reporting on race, ethnicity, or other socially relevant groupings	*Please specify the socially constructed or socially relevant categorization variable(s) used in your manuscript and explain why they were used. Please note that such variables should not be used as proxies for other socially constructed/relevant variables (for example, race or ethnicity should not be used as a proxy for socioeconomic status). Provide clear definitions of the relevant terms used, how they were provided (by the participants/respondents, the researchers, or third parties), and the method(s) used to classify people into the different categories (e.g. self-report, census or administrative data, social media data, etc.) Please provide details about how you controlled for confounding variables in your analyses.*
Population characteristics	*Describe the covariate-relevant population characteristics of the human research participants (e.g. age, genotypic information, past and current diagnosis and treatment categories). If you filled out the behavioural & social sciences study design questions and have nothing to add here, write "See above."*
Recruitment	*Describe how participants were recruited. Outline any potential self-selection bias or other biases that may be present and how these are likely to impact results.*
Ethics oversight	*Identify the organization(s) that approved the study protocol.*

Note that full information on the approval of the study protocol must also be provided in the manuscript.

Field-specific reporting

Please select the one below that is the best fit for your research. If you are not sure, read the appropriate sections before making your selection.

☒ Life sciences ☐ Behavioural & social sciences ☐ Ecological, evolutionary & environmental sciences

For a reference copy of the document with all sections, see nature.com/documents/nr-reporting-summary-flat.pdf

Life sciences study design

All studies must disclose on these points even when the disclosure is negative.

Sample size	A statistician not involved in the study calculated the sample size prior to the experiment. The sample size was determined based on comparisons between infected animals and a control group at seven time points, with different animals tested at each point, resulting in 28 independent comparisons. The study aimed to detect a 10% difference between group means with a standard deviation of 4%, 80% power, and a 5% trial-wide error rate adjusted using the Bonferroni method ($\alpha = 0.001785$). Using the PASS 2021 program, the minimum required sample size was calculated as 8 animals per group.
Data exclusions	all obtained data were included
Replication	the animal experiment, immunolabellings, quantifications, and data analysis were perfomed once

Randomization	allocation to experimental groups was randomized
Blinding	the investigators were not blinded to group allocation during the animal experiment for obvious reasons due to BSL3 safety conditions to avoid cross-infections. for morphologic analysis samples were blinded before examination.

Reporting for specific materials, systems and methods

We require information from authors about some types of materials, experimental systems and methods used in many studies. Here, indicate whether each material, system or method listed is relevant to your study. If you are not sure if a list item applies to your research, read the appropriate section before selecting a response.

Materials & experimental systems

n/a	Involved in the study
☐	☒ Antibodies
☐	☒ Eukaryotic cell lines
☒	☐ Palaeontology and archaeology
☐	☒ Animals and other organisms
☒	☐ Clinical data
☒	☐ Dual use research of concern
☒	☐ Plants

Methods

n/a	Involved in the study
☒	☐ ChIP-seq
☒	☐ Flow cytometry
☒	☐ MRI-based neuroimaging

Antibodies

Antibodies used

CK8 (Invitrogen PA-29607) polyclonal, rabbit, 1 : 500
CK8-Alexa Fluor 488 conjugated (abcam, AB192467) monoclonal, rabbit, clone EP1628Y, 1 : 100
SCGB1A1 (Proteintec, 10490-1-AP) polyclonal, rabbit, 1 : 1600
SCGB1A1-Alexa Fluor 488-conjugated (Proteintec CL488-10490) polyclonal, rabbit, 1 : 1600
CK14 (invitrogen, PA5-16722) polyclonal, rabbit 1 : 500
CK14 (invitrogen, MA5-11599) monoclonal, mouse, clone LL002, 1 : 250
proSP-C (MEMD Millipore, AB3786) polyclonal, rabbit, 1 :1000
Pdpn (kindly provided by Prof. Kato, Tohoku University, Japan) monoclonal, mouse, 281-mG2a-f, 1 : 400
p53 (NovusBiologicals, Corporation, MAB1710) monoclonal, mouse, clone SRA-E5, 1 : 200
P53-12) monoclonal, mouse, clone BP53-12, 1 : 200
Ki-67 (abcam, ab15580-100) polyclonal, rabbit, 1 : 500
p21 (SantaCruz Biotechnology, sc6246) monoclonal, mouse, F-5, 1 : 100 IHC, 1 : 1000 IF
Δnp63 (Cell Signaling #67825) monoclonal, rabbit, clone E6Q30 1 : 1000
CD3 (Dako, A0452) polyclonal, rabbit, 1 : 500
Pax5 (biolegend Cat649702) monoclonal, rat, 1H9, 1 : 500
Iba-1 (ThermoFisher, PA5-27436) polyclonal, rabbit , 1 : 1000
MPO (abcam, ab9535) polyclonal, rabbit, 1 : 200
Plet-1 (biorbyt, orb312797_1) polyclonal, rabbit, 1 : 200
CD204 (Abnova Corporation, MAB1710) monoclonal, mouse, clone SRA-E5, 1 :500
SARS-CoV-2 NP (Sinobiological, 40143-MM05) monoclonal, mouse, clone 5, 1 : 16000
SARS-CoV-2 SP (Sino Biological Inc., HD14AP0706) polyclonal, rabbit, 1 : 4000

Validation

all antibodies were used follwoing the manufactureres recommendations. antiboides were validated for at least immunohistochemistry/fluorescence or immunoblotting by the manufacurer. hamster specie reactivity was ensured for the majority of the antibodies. All antibodies ensured reactivity for human and mice. we indeed tested for reactivity in different target organs for the investigated protein using mouse and hamster tissues in parallel and confirming antibodies specificity for the target

Eukaryotic cell lines

Policy information about cell lines and Sex and Gender in Research

Cell line source(s)	Vero cells (ATCCR, CRL-1586)
Authentication	all cell lines used were purchased as stated above but not indipendently authenticated
Mycoplasma contamination	all cells used were mycoplasma free
Commonly misidentified lines (See ICLAC register)	no commonly misidentified cells were used

Animals and other research organisms

Policy information about studies involving animals; ARRIVE guidelines recommended for reporting animal research, and Sex and Gender in Research

Laboratory animals	syrian golden hamsters (RjHan:AURA, 11-12 month old) purchased from Janvier Labs, France, the strain originates from Zentralinstitut für Versuchstierzucht (Hannover) - 1990
Wild animals	the study did not include wild animals
Reporting on sex	only male hamsters were used. rationale: For this study we selected the sex and age group (1 year old male hamsters) that is more likely to develop respiratory long-term consequences of SARS-CoV-2 infection
Field-collected samples	field-collected samples were included in this study
Ethics oversight	The animal experiment was in accordance with the EU directive 2010/63/EU and approved by the state office for consumer protection and food safety of Lower Saxony (Niedersächsisches Landesamt für Verbraucherschutz und Lebensmittelsicherheit, LAVES (protocol code TV22-00088, approval 16.09.2022).

Note that full information on the approval of the study protocol must also be provided in the manuscript.

Plants

Seed stocks	*Report on the source of all seed stocks or other plant material used. If applicable, state the seed stock centre and catalogue number. If plant specimens were collected from the field, describe the collection location, date and sampling procedures.*
Novel plant genotypes	*Describe the methods by which all novel plant genotypes were produced. This includes those generated by transgenic approaches, gene editing, chemical/radiation-based mutagenesis and hybridization. For transgenic lines, describe the transformation method, the number of independent lines analyzed and the generation upon which experiments were performed. For gene-edited lines, describe the editor used, the endogenous sequence targeted for editing, the targeting guide RNA sequence (if applicable) and how the editor was applied.*
Authentication	*Describe any authentication procedures for each seed stock used or novel genotype generated. Describe any experiments used to assess the effect of a mutation and, where applicable, how potential secondary effects (e.g. second site T-DNA insertions, mosiacism, off-target gene editing) were examined.*
